# Successful mitral-transcatheter edge to edge repair in a young patient with severe primary mitral regurgitation unsuitable for surgery: a case report

**DOI:** 10.1093/ehjcr/ytaf464

**Published:** 2025-09-19

**Authors:** Giovanni Bellina, Salvatore Scandura, Carmelo Grasso, Placido Maria Mazzone, Davide Capodanno

**Affiliations:** Cardiology Unit, Azienda Ospedaliera Universitaria Policlinico ‘G. Rodolico-San Marco’, Via Santa Sofia n° 78, Padiglione 8, Catania 95125, Italy; Cardiology Unit, Azienda Ospedaliera Universitaria Policlinico ‘G. Rodolico-San Marco’, Via Santa Sofia n° 78, Padiglione 8, Catania 95125, Italy; Cardiology Unit, Azienda Ospedaliera Universitaria Policlinico ‘G. Rodolico-San Marco’, Via Santa Sofia n° 78, Padiglione 8, Catania 95125, Italy; Cardiology Unit, Azienda Ospedaliera Universitaria Policlinico ‘G. Rodolico-San Marco’, Via Santa Sofia n° 78, Padiglione 8, Catania 95125, Italy; Cardiology Unit, Azienda Ospedaliera Universitaria Policlinico ‘G. Rodolico-San Marco’, Via Santa Sofia n° 78, Padiglione 8, Catania 95125, Italy

**Keywords:** Mitral transcatheter edge-to-edge repair, Primary mitral regurgitation, Young patient, Transoesophageal echocardiogram, Case report

## Abstract

**Background:**

Mitral transcatheter edge-to-edge repair (M-TEER) is commonly performed in elderly patients with severe mitral regurgitation (MR) who are deemed high-risk for surgery. However, its application in younger patients remains limited.

**Case summary:**

We present the case of a 45-year-old male with a history of mitral valve prolapse, who developed severe MR due to chordal rupture. Surgical mitral valve repair was not considered feasible, and the patient successfully underwent M-TEER with implantation of two MitraClips XTW (Abbott Medical, USA), resulting in mild residual regurgitation and a mean mitral valve gradient of 5 mmHg.

**Discussion:**

This case highlights the potential role of M-TEER in selected young patients, expanding its indications beyond traditional cohorts.

Learning points

- Mitral transcatheter edge-to-edge repair is a viable option for younger patients with severe primary mitral regurgitation when surgical repair is not feasible. This case highlights the need for further studies to assess long-term outcomes in younger populations undergoing transcatheter mitral repair.

## Introduction

Mitral regurgitation (MR) is the second most frequent valvular heart disease in Europe,^1^ and mitral transcatheter edge-to-edge repair (M-TEER) has emerged as a minimally invasive alternative to surgery in patients deemed at high surgical risk.^2^ While M-TEER is well established in elderly populations, its role in younger patients, particularly those with primary MR due to chordal rupture, remains controversial.^3^ This case report describes a successful M-TEER procedure in a young patient with severe MR highlighting its feasibility in this patient subgroup.

## Summary figure

**Figure:**
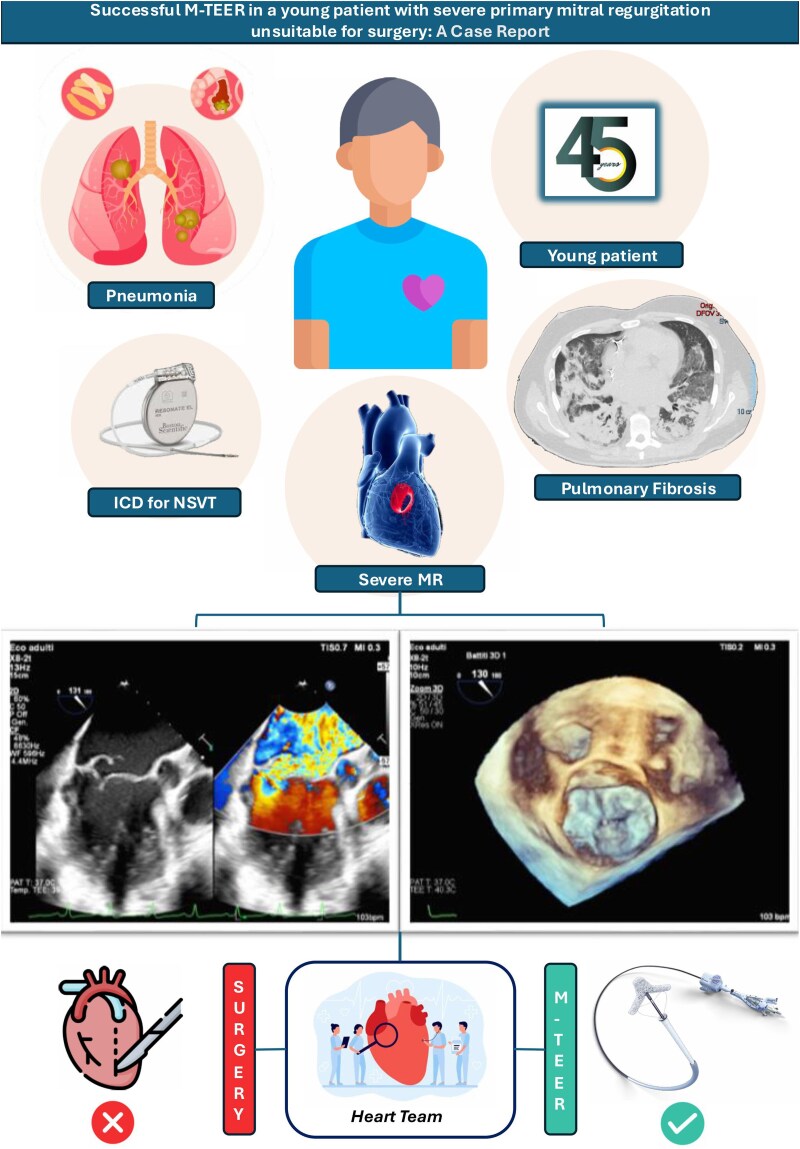
Patient history, risk factor, and Heart Team decision for M-TEER; pre-procedural transoesophageal echocardiography images including LVOT views and 3D images; M-TEER, transcatheter edge-to-edge mitral valve repair.

## Case presentation

A 45-year-old male was admitted in February 2025 due to dyspnoea and fever. He was a former smoker and suffered from hypertension. In 2011, following episodes of palpitations and the detection of occasional premature ventricular contractions on a routine ECG, he underwent an exercise ECG which was terminated due to non-sustained ventricular arrhythmia. The diagnostic evaluation proceeded with a transthoracic echocardiogram (TTE) that showed mild mitral valve regurgitation due to lealflets’ billowing and normal left ventricular ejection fraction (55%). Thereafter, coronary angiography ruled out ischaemic heart disease and cardiac MRI excluded arrhythmogenic right ventricular dysplasia and left ventricular non-compaction. Genetic testing ruled out known mutations linked to arrhythmogenic cardiomyopathy.

Finally, a loop recorder was implanted which revealed episodes of non-sustained polymorphic ventricular tachycardia, leading to the decision to implant an ICD for primary prevention.

Between 2012 and 2024, the patient remained clinically stable.

**Figure 1:**
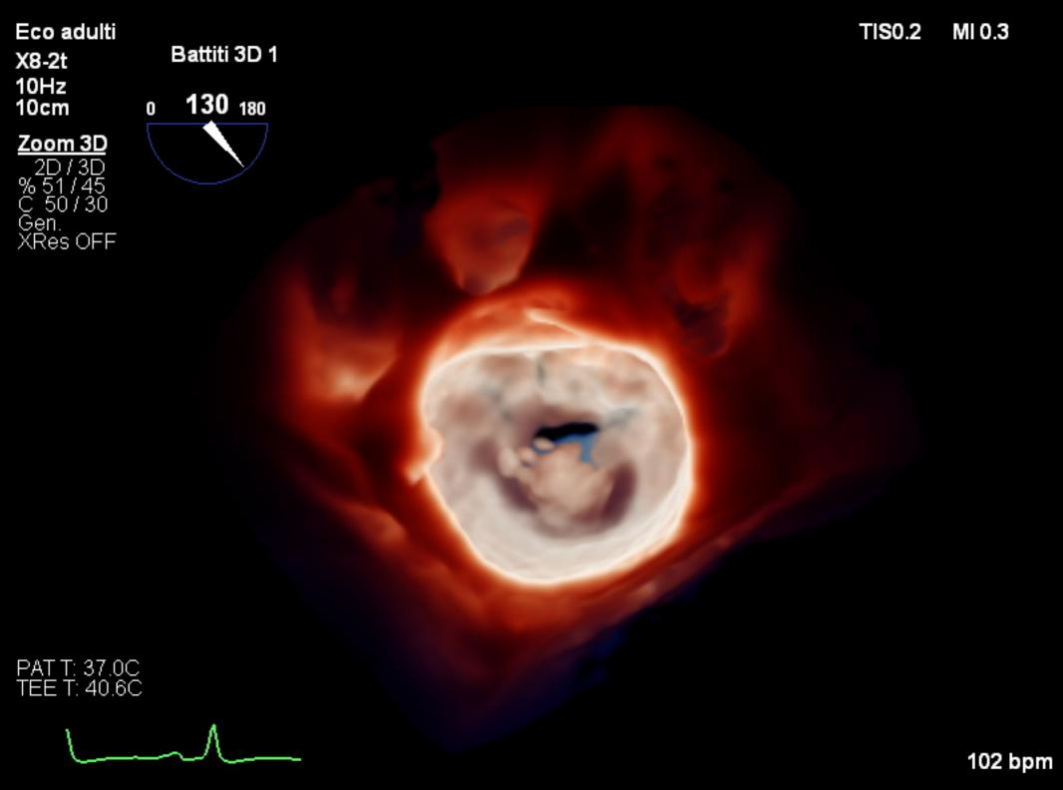
TEE mitral valve in 3D Touchview modality, left atrial view.

**Figure 2:**
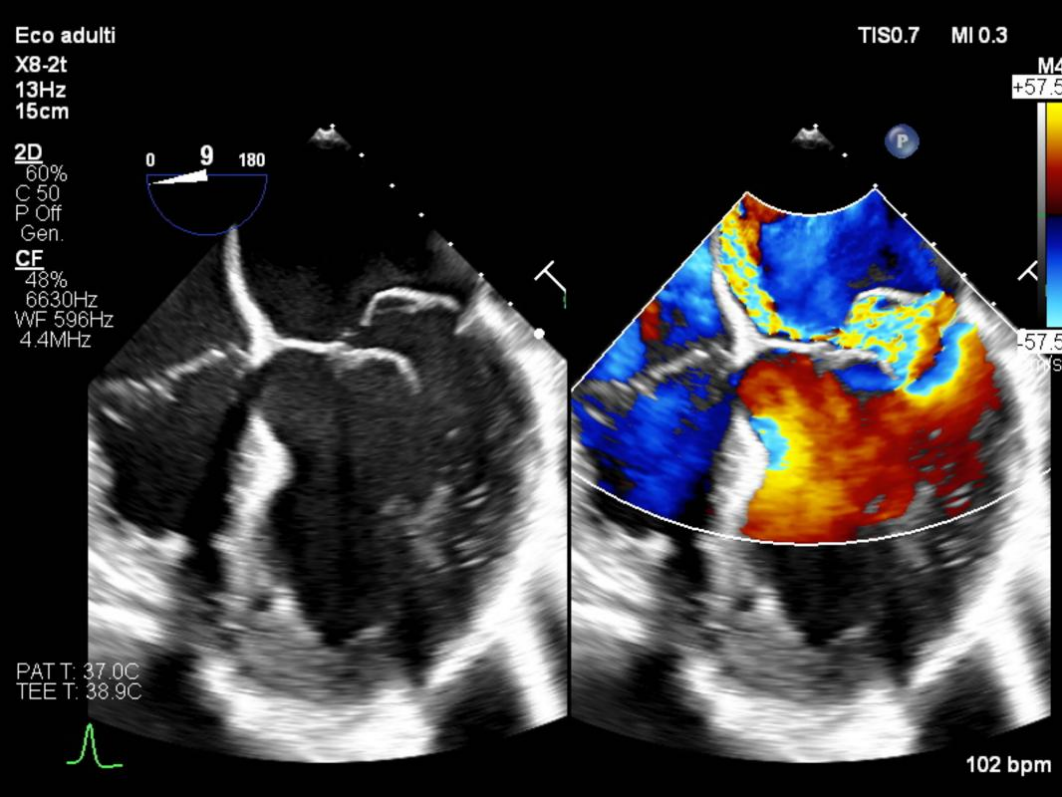
TEE 4CH, mitral valve with and without colour, Coanda effect.

In November 2024, during a routine follow-up visit, an echocardiogram showed moderate MR due to mitral valve prolapse. In February 2025, as noted, he was admitted due to dyspnoea and fever. The patient appeared tachypnoeic, and the auscultation revealed bilateral fine inspiratory crackles (‘Velcro-like’), especially at the lung bases. On cardiac examination, a holosystolic murmur was heard at the apical area. Abdominal examination was normal. Blood pressure was 100/60 mmHg, heart rate 95 beats per minute, and the respiratory rate 20 breaths per minute. He underwent blood tests which showed an increase in inflammatory indices [WBC 25.5 × 10^3^ ηg/L (n.r. 4.5–11 × 103 ηg/L), C-reactive protein 45 mg/dL (n.r. 0.3–1 mg/dL), ESR 118 mm/h (0–15 mm/h)] but normal liver and kidney function. ECG showed non-specific ventricular repolarization abnormalities. Chest X-ray demonstrated multiple confluent opacities in the mid-right lung field. The TTE and transoesophageal echocardiogram (TEE) showed large flail of the mitral valve posterior leaflet (P2, *Figure 1*; Supplementary material online, *Video S1*) due to chordal rupture, with a 12 mm coaptation gap and an eccentric regurgitant jet exhibiting a Coanda effect—consistent with severe MR. (*Figure 2*; Supplementary material online, *Video S2*). The posterior leaflet measured 10 mm in length. The left atrium was dilated; pulmonary artery systolic pressure was estimated at 40 mmHg. LVEF was preserved at 52%.

Chest CT showed thickened interlobular septa with pseudo-nodular consolidations and diffuse reticular thickening of the peribronchovascular interstitium (see Supplementary material online, *Figure S1*; Supplementary material online, *Video S3*). A presumptive diagnosis of pulmonary fibrosis complicated by pneumonia was made.

Pulmonary fibrosis exhibited a non-specific interstitial pneumonia pattern.

The patient was treated with non-invasive ventilation, intravenous methylprednisolone (20 mg/day), and antibiotics (linezolid 600 mg b.i.d., meropenem 500 mg t.i.d.). Despite medical therapy, the patient remained haemodynamically unstable due to severe MR and required inotropic support.

In March 2025, the patient was transferred to the cardiac surgery unit. The Heart Team determined that ‘although surgical therapy is the first choice in a young patient with acute severe MR, the mitral valve could not be surgically replaced due to the severity of the pulmonary condition. Therefore, M-TEER was selected as the preferred treatment option’.

**Figure 3:**
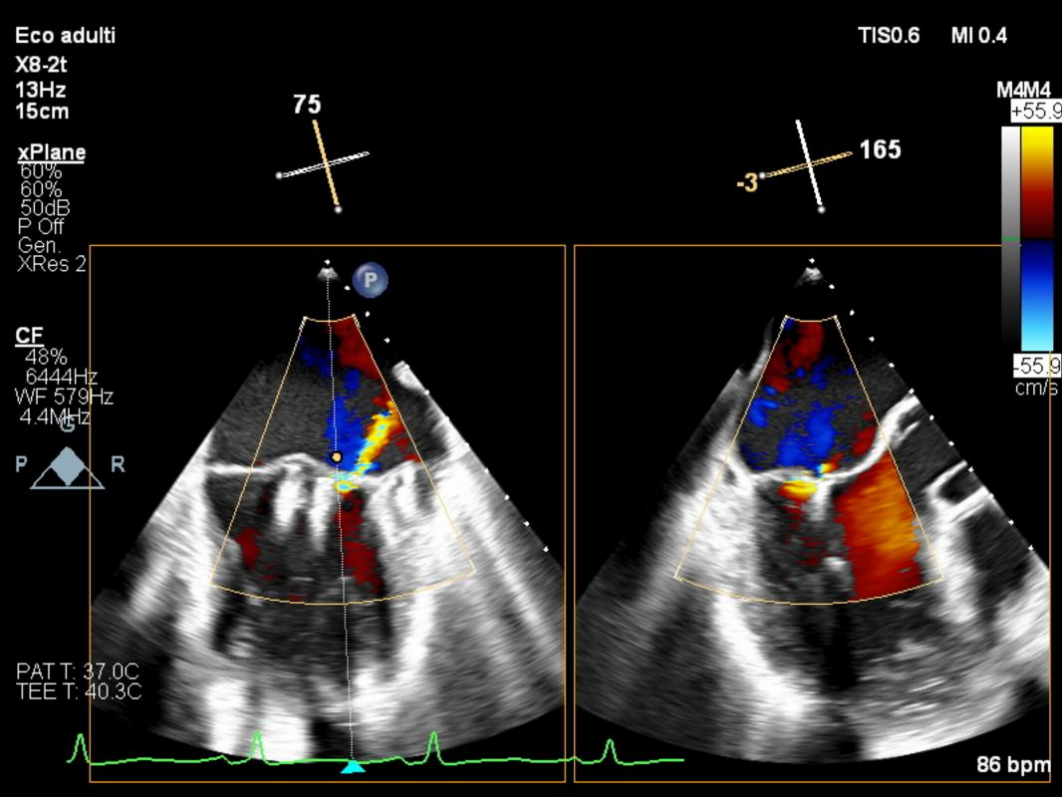
TEE xplane colour placement clip XTW n°2.

**Figure 4:**
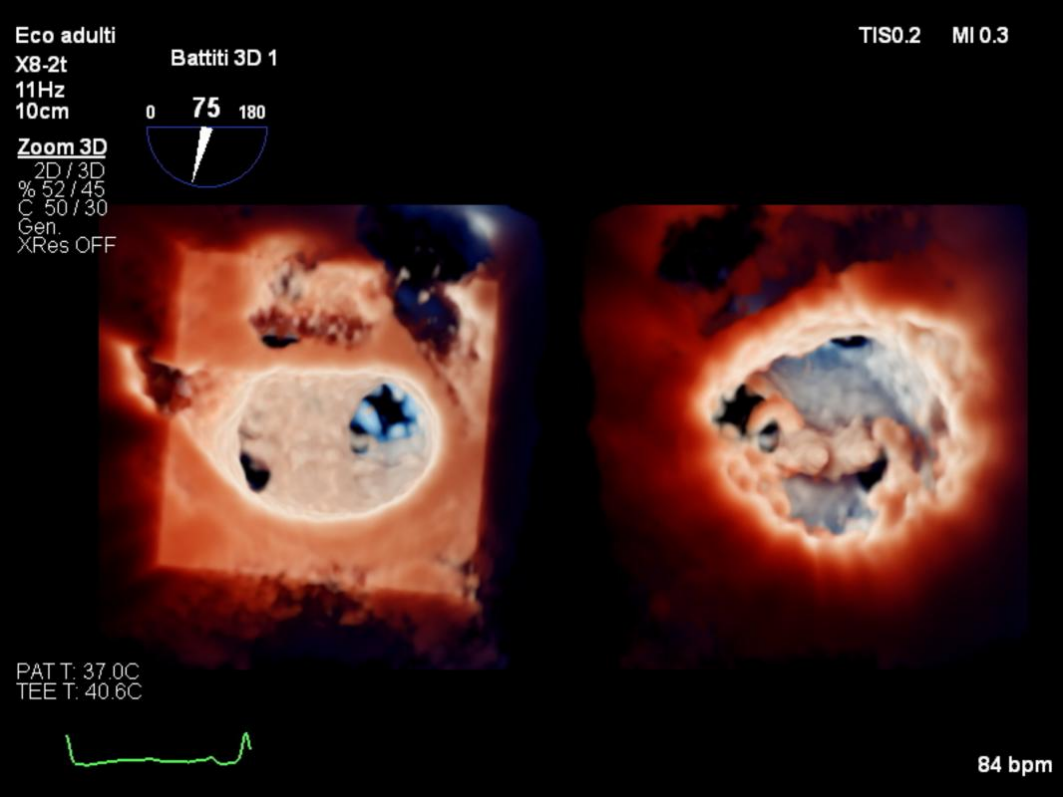
TEE mitral valve with n°2 clip XTW in 3D Touchview, left atrium and ventricular view.

On 5 March 2025, the M-TEER procedure was performed under general anaesthesia with fluoroscopic and transoesophageal echocardiographic guidance. Following right femoral vein puncture, a 12 Fr introducer was positioned. A Mullins catheter was advanced, and transseptal puncture was performed using a Brockenbrough needle. The transseptal puncture was performed in a posterior and inferior position to avoid the aorta and to achieve an adequate height—∼4 cm from the mitral valve plane—allowing sufficient angulation to manoeuvre and direct the catheter towards the valve. The Mullins catheter was then advanced into the left atrium and selectively into the left superior pulmonary vein. Proper catheter positioning was confirmed via contrast injection, and an Amplatz Super Stiff guidewire was introduced into the left superior pulmonary vein. The guiding catheter system was advanced into the left atrium, followed by the introduction of the clip delivery system. A first XTW clip was deployed at the A2–P2 segment, resulting in moderate and lateral residual MR (mean gradient 2 mmHg) (see Supplementary material online, *Figure S2*; Supplementary material online, *Video S4*). A second XTW clip was then implanted laterally to the first, achieving mild residual regurgitation (mean gradient 4 mmHg) (*Figure 3*; Supplementary material online, *Video S5A*–*C*). Angiographic control confirmed the absence of contrast extravasation from the left femoral artery. The right femoral venous access was closed using two ProGlide devices, while the left femoral arterial introducer was removed in the procedure room, and haemostasis was achieved using the FemoSeal system. The procedure was successful and without complications (*Figure 4*; Supplementary material online, *Video S6*).

Ten days after M-TEER procedure, the patient’s clinical condition was improving, with no ventilation or inotropic support needed. A follow-up echocardiogram showed sustained procedural success (see Supplementary material online, *Video S7*). He was discharged 15 days post-procedure with full clinical recovery.

## Discussion

This case highlights the potential role of M-TEER as an alternative to surgery in selected younger patients with degenerative MR. Here, the severity of the lung condition (fibrosis and pneumonia) negatively affected the surgical risk (patient’s Society of Thoracic Surgeons combined morbidity and mortality score was 34%), making conventional surgery unfeasible.

Although durability concerns and the possibility of reintervention remain limitations of M-TEER in younger individuals, it offers a viable, minimally invasive option when surgery is contraindicated.

## Conclusion

Mitral transcatheter edge-to-edge repair represents a feasible and effective therapeutic strategy in younger patients with severe primary MR when surgery is not an option. Further studies are warranted to evaluate the long-term outcomes of transcatheter mitral repair in this population.

## Lead author biography


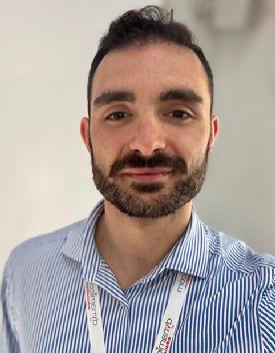


Giovanni Bellina is a medical doctor, currently a Cardiology resident at Catania’s University in Italy. He attended the University of Catania where he graduated with honours in 2020. He is passionate about clinical and cardiovascular imaging; he attends assiduously the cardiological intensive care unit and the cardiovascular imaging clinic of C.A.S.T.-Policlinico Hospital in Catania.

## Supplementary Material

ytaf464_Supplementary_Data

## Data Availability

The data underlying this article are available in the article and in its online Supplementary material.
