# Supplementary figures and images for: Successful mitral-transcatheter edge to edge repair in a young patient with severe primary mitral regurgitation unsuitable for surgery: a case report
Source: Eur Heart J Case Rep. 2025 Sep 19;9(10):ytaf464. doi: 10.1093/ehjcr/ytaf464 (PMC12495034; doi:10.1093/ehjcr/ytaf464)

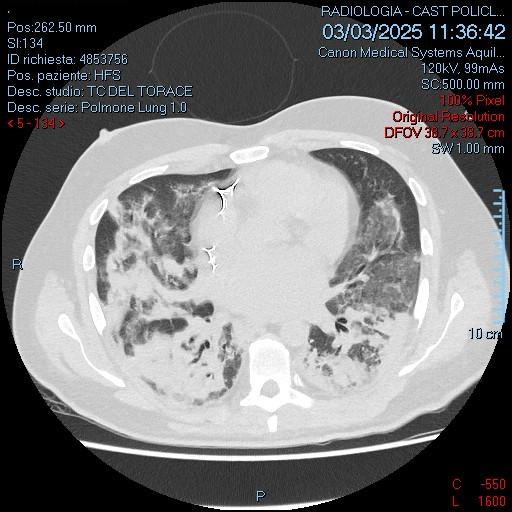

Supplement: ytaf464_Supplementary_Data [file ytaf464_supplementary_data.zip › Supplementary figure 1. Chest CT v2 .tiff]

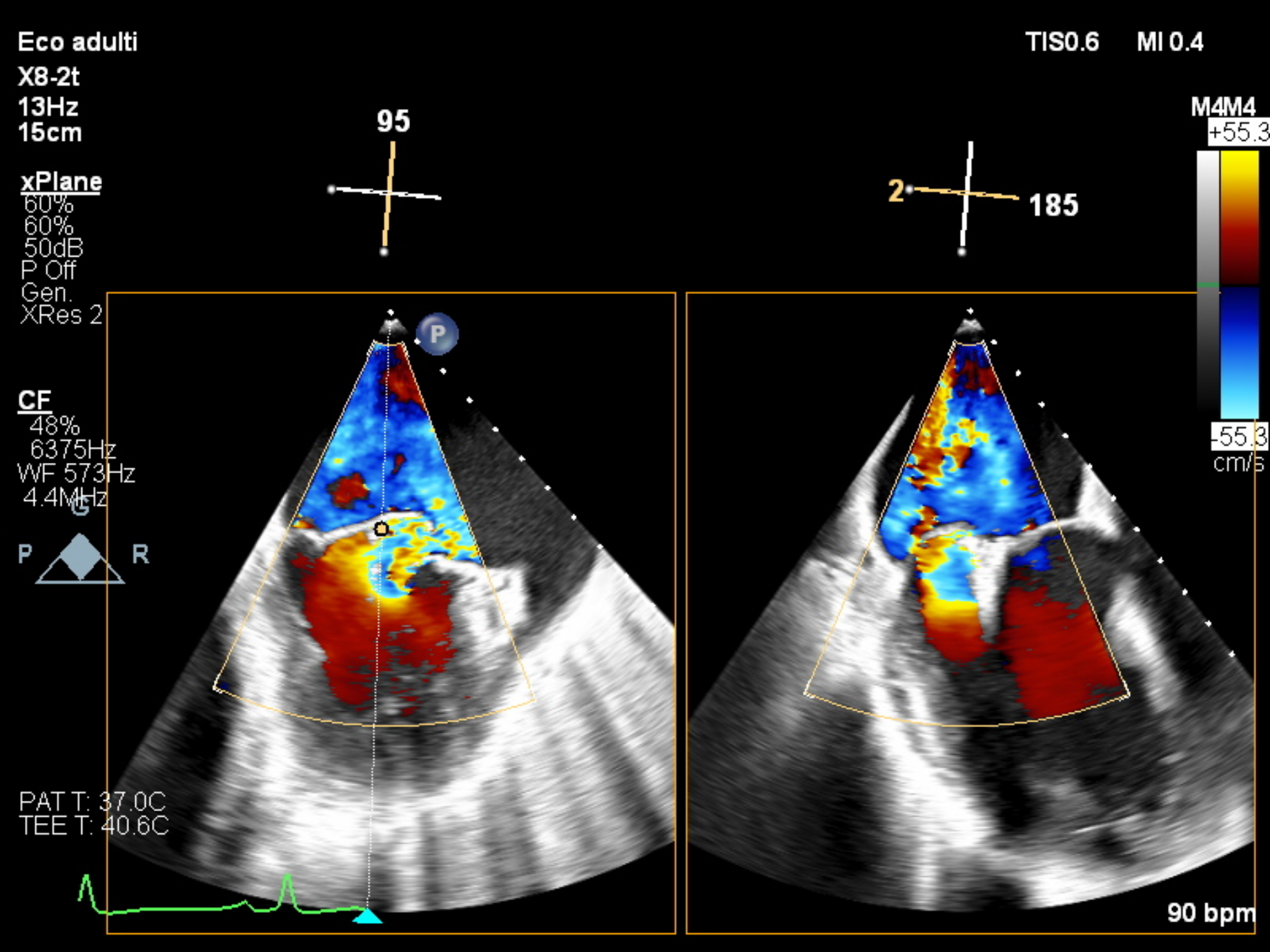

Supplement: ytaf464_Supplementary_Data [file ytaf464_supplementary_data.zip › Supplementary figure 2.tif]
